# Comparative Studies in Tick-Borne Diseases in Animals and Humans

**DOI:** 10.3390/vetsci4020032

**Published:** 2017-06-20

**Authors:** Ulrike Munderloh

**Affiliations:** Department of Entomology, University of Minnesota, 1980 Folwell Avenue, St. Paul, MN 55108, USA; munde001@umn.edu

In temperate zones of the earth, ticks are the most important arthropod vectors of zoonotic pathogens affecting humans and domestic animals. Ticks transmit the widest range of pathogen types, from viruses to rickettsiae, other bacteria, and protozoa. Historically, research with tick-borne pathogens has lagged behind research with mosquitoes, owing to a combination of their much longer life-cycle spanning months even under laboratory conditions, the requirement for multiple blood meals, and lack of standardized rearing systems. This issue presents a collection of original and review articles of the most up-to-date research and perspectives in the field. It highlights the complex life cycle strategies tick-borne disease agents have evolved to take advantage of their extraordinarily divergent hosts in order to facilitate continued circulation in nature. Importantly, emerging tick-borne pathogens are commonly zoonotic; they are maintained in complex cycles involving wild reservoir hosts and ticks, and are transmitted to humans and domestic animals as incidental hosts. The development and application of innovative technologies has spurred advances that address long-standing questions about the behavior of pathogenic and non-pathogenic symbiotic bacteria in ticks, and enable modelling of arbovirus development in tick cell and organ cultures. Cell culture systems have long been indispensable tools in medical research, and are amenable to sophisticated molecular and cell-biological approaches to dissect the pathogenesis of obligate intracellular bacteria, many of which are associated with ticks. Cell lines from a number of tick species important in human and veterinary medicine are increasingly utilized to address these questions, as highlighted by three articles in this special issue. Unlike rickettsiae that are in direct contact with host cytoplasm, *Anaplasmatceae* are sheltered from host defenses inside a membrane-bound compartment, and have evolved mechanisms to accommodate their needs. The article by Crowder and colleagues [1] provides new insights into how *A. phagocytophilum* remodels that barrier to ensure protection, flow of nutrients, and availability of conduits for the secretion of effectors to control host cell functions. The intracellular niche occupied by *A. phagocytophilum* provides unique opportunities to uncover novel cell defense signaling pathways that cannot be identified using facultatively intracellular bacteria, as reviewed by Shaw and colleagues in this issue [2]. 

Whole genome sequencing of tick-borne bacteria has accelerated the study of microbial ecology and host adaptation to yield a detailed picture of how bacterial communities across species that utilize the same generalist vector form similar patterns of host utilization to maximize host availability. At the same time, detailed molecular analyses have begun to illuminate bacterial signatures that underlie pathogenicity and immunogenicity, guiding the development of therapeutics and vaccines. Nevertheless, a number of tick-borne pathogens have surprisingly sophisticated capabilities to evade the mammalian immune response, and to remain either undetected or cause overwhelming infections. Thus, many still pose challenges for diagnosis. Moreover, *Borrelia* spp. that infect a variety of vertebrates have evolved mechanisms that enable them to utilize species-specific features of complement pathways to infect their many hosts [3]. Clearly, tick-borne zoonotic bacteria communicate extensively with the infected vertebrates, responding with adaptive changes as well as by active manipulation of hosts.

Far less research has focused on the effects of the pathogens on their tick vector. It is generally assumed that midgut and salivary glands are target organs in the tick, as demonstrated using PCR-based detection of dissected tissues. From those limited investigations, it is clear that *A. phagocytophilum* recognizes these different target organs as distinct as evident from their apoptosis signaling and pathway responses (see Alberdi et al. [4] in this issue). This specificity is also reflected in the divergent regulation of apoptosis pathways in tick cell lines, which makes them useful models to study tick immune responses.

Hard and soft ticks and their associated pathogens are spread widely across the globe, yet recognition of their presence and disease incidence is linked to the amount of scientific scrutiny expended. Globally, regions where ticks serve as vectors of zoonotic pathogens will continue to yield records of the range expansion of known pathogens and of the discovery of newly recognized agents. Thus, the field of tick-borne zoonotic disease agents is continuously changing and far from static. This issue represents a snapshot of our current understanding, and is meant to stimulate discussion to advance all aspects of this fascinating subject.
